# Dextrose Prolotherapy to Treat Pain, Improve Activities of Daily Living, and Improve Quality of Life in an Ewing's Sarcoma Patient Following Radiation and Chemotherapy Treatment

**DOI:** 10.7759/cureus.13549

**Published:** 2021-02-25

**Authors:** Andre Panagos

**Affiliations:** 1 Rehabilitation Medicine, New York University (NYU) Grossman School of Medicine, New York, USA; 2 Rehabilitation Medicine, New York City (NYC) Health + Hospitals/Bellevue, New York, USA; 3 Physical Medicine and Rehabilitation, Spine & Sports Medicine of New York, New York, USA

**Keywords:** prolotherapy, ewing's sarcoma, pelvic pain, chronic pain management, chronic low back pain (clbp), dextrose prolotherapy, refractory cancer pain, chronic post surgical pain, regenerative medicine therapies, regenerative procedures

## Abstract

Advances in the treatment and survival of Ewing’s sarcoma patients create a need to treat underlying symptoms that limit activities of daily living and quality of life. This case describes the treatment of pain in a 25-year-old female pediatric nurse with Ewing’s sarcoma of the pelvis that was in remission following radiation and chemotherapy. She reported medication side effects and limitations in her activities of daily living and quality of life with the chronic use of topical and oral pain medications. A dextrose prolotherapy approach was used to treat her pain, which allowed her to discontinue her pain medication regimen, resulting in an improvement in her activities of daily living and quality of life. The improvement was sustained at the three-year follow-up after the last procedure.

## Introduction

Ewing sarcoma (ES) is the most common primary malignant bone tumor of the pelvis in children and adolescents [1]. Up to 25% of ES cases originate in the pelvis, representing approximately 8% of all primary malignant bone tumors [2-3]. Survival has greatly improved in the past three decades due to advances in treatment, which are most frequently resection and radiation. ES of the pelvis continues to have a poor prognosis as compared to other sites due to the ease of tumor migration into the adjacent abdominal organs and local neurovascular bundles [4]. Patients with primary tumors of the pelvis have the greatest risk of long-term impairments, especially if they take prescription pain medications and have decreased exercise frequency [5].

ES characteristically presents with pain that is worse at night with localized swelling. Symptoms can also include fatigue, weakness, numbness, and paralysis. Injury or fracture due to structural weakness is usually the first diagnostic clue. Imaging commonly notes an aggressive lesion, which only occasionally presents with the classic “onion peel” appearance [6]. Chronic pain management is used to mitigate the effects of pain on activities of daily living and quality of life following treatment.

The treatment of non-carcinogenic posterior pelvic pain is fraught with uncertainties as to the true diagnosis. Most treatments are centered around the sacroiliac joint and its innervation. Treatments include injection of corticosteroids into the sacroiliac joint and sacroiliac joint radiofrequency rhizotomy. Regenerative injections include dextrose prolotherapy (DPT), platelet-rich plasma, and stem cells. Research has found that the treatment of sacroiliac joint pain with CT-guided DPT can decrease sacroiliac joint instability and decrease pain up to 24 months [7-8].

## Case presentation

This case describes the history and treatment course of a 25-year-old, full-time pediatric nurse who presented to a private clinic in 2012 with right greater than left low back and sacral pain that radiated to her right lateral foot. She reported that her pain complaint began in 2004 with an initial diagnosis of pelvic pain related to her menstrual cycle. As her pain worsened, she tried chiropractic care and muscle relaxants with no improvement. She then began to notice a bulge in her right sacrum, yet X-rays remained normal. By the time she graduated from high school and started college one year later, her pain began to interrupt her sleep and she developed significant weakness in her right lower extremity, resulting in a significant gait abnormality. A CT scan reported abnormal findings in the right sacral ala. Further testing included an MRI with contrast, a needle biopsy, and an open surgical biopsy, which confirmed ES. The patient reviewed her options and did not opt for a full resection due to the risk of an impaired or total loss of bowel and bladder function, limited use of her right lower extremity, and diminished quality of life. She instead chose to be treated with chemotherapy and radiation. The swelling and right lower extremity weakness gradually improved after treatment, yet her pain did not improve. A remission was confirmed based on annual surveillance imaging before she presented to our clinic.

**Figure 1:**
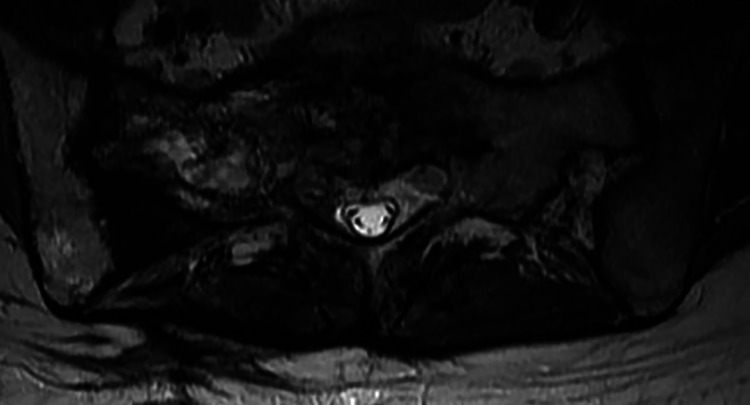
Axial T2-weighted fast recovery fast spin-echo (FRFSE) MRI of the pelvis Axial T2-weighted fast recovery fast spin-echo (FRFSE) MRI of the pelvis demonstrating an ill-defined lesion representing Ewing's sarcoma within the sacrum (red arrows). The lesion is eccentric to the right involving a large portion of the right sacral ala interspersed with fat, which is compatible with areas of bone infarction. The site of the open surgical biopsy within the right posterior lumbar paraspinal and subcutaneous tissues (blue arrows) is also the site of the posterior sacroiliac joint ligament.

**Figure 2:**
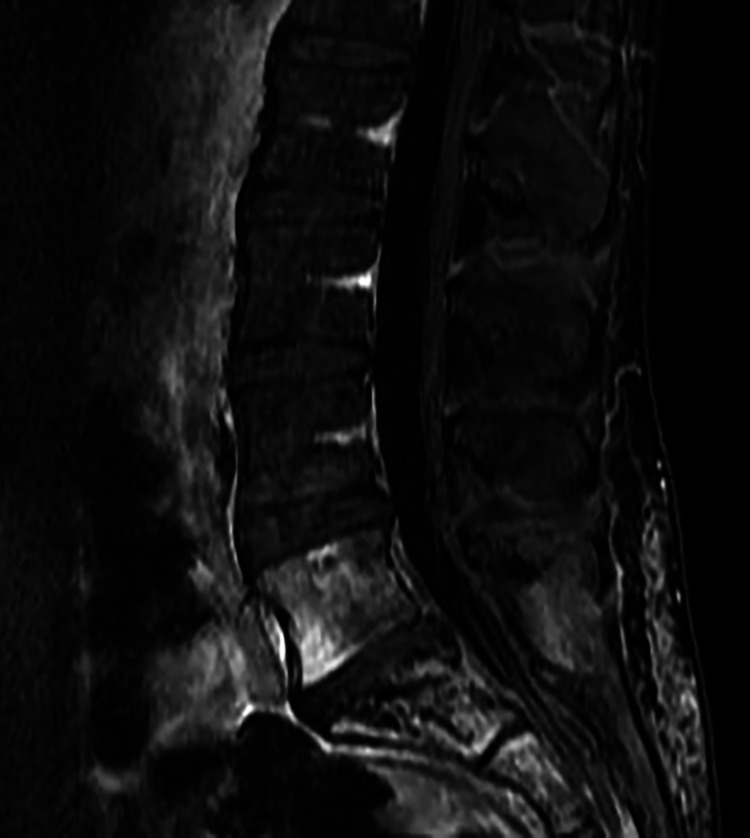
Sagittal T1-weighted fluid-attenuated inversion recovery (FLAIR) MRI with contrast of the lumbosacral junction Sagittal T1-weighted fluid-attenuated inversion recovery (FLAIR) MRI with contrast of the lumbar spine demonstrating fatty replacement at the L5 vertebral body and sacrum (red arrows) with postoperative scarring within in the subcutaneous tissues, paraspinal muscles, and sacrum (blue arrows).

On her first visit in 2012, she reported that her pain was visual analog scale (VAS) 8-9/10 without pain medications and VAS 3-4/10 with pain medications. She felt that her pain medication regimen allowed her to accomplish the basic activities of daily living and occupational activities. She was very hesitant to engage in physical or recreational activities. Her past medical history was only pertinent for asthma. Her pain medication regimen was pregabalin 50 mg PO bid, a fentanyl patch 75 mcg q48h, and oxycodone 15mg, ½ - 1 PO q4-6hrs prn (2/day). Zofran was used as needed for nausea. A follow-up MRI of the lumbar spine in May 2014 noted an ill-defined mass-like lesion within the sacrum (Figure 1) and fatty replacement of the L5 vertebral body and sacrum (Figure 2).

The physical examination was pertinent for tenderness at and surrounding the incision site at the right lumbosacral region as well as the surrounding right posterior superior iliac spine region. Sacroiliac joint provocative maneuvers were positive on the right. She demonstrated no tenderness at the posterior hip capsule or upper lumbar paraspinal. Excellent hip range of motion was noted bilaterally. Straight leg raise was negative. Lower extremity strength, sensation, and tone were normal. Reflexes were absent at the bilateral ankles and right knee.

The initial clinical diagnosis included sacroiliac joint pain/instability in addition to fibrotic incisional scar tissue due to the open surgical biopsy and radiation. Between January 19, 2015, and May 12, 2017, 20 sessions of fluoroscopically guided DPT targeted the right L4/5 and L5/S1 facet joints, the L4-S2 supraspinous and interspinous ligaments, the right posterior sacroiliac ligament, the right sacrospinous ligament, the right iliolumborum ligament, and the right gluteus maximus/medius/minimus tendon origins at the right iliac crest using up to 60 ml of a 25% dextrose solution with 10 ml 1% lidocaine. Initially, the patient demonstrated minimal improvement, but increasingly, she demonstrated greater lasting improvement that became centralized just lateral to the healed incision site at the right lateral posterior superior iliac spine.

Improvement was also evidenced with decreasing pain medication requirements. Pregabalin was discontinued on November 15, 2015. The long-acting fentanyl patch was slowly titrated down and discontinued as well. She reported that after each dextrose prolotherapy session, she would have a two-week exacerbation of her pain, which required temporarily greater pain medication use. By January 8, 2016, she reported taking only one to two tablets of oxycodone per day. At her June 15, 2017 visit, the patient reported that she did not need further pain medication refills. She reported that her pain level was VAS 0-2/10 without pain medications. Her physical examination was pertinent for resolution of local tenderness at the incision site and minimal pain with sacroiliac joint provocative maneuvers. Her activities of daily living and occupation were reported to be much easier to accomplish. Her last office visit was July 24, 2017. In December 2020, the patient was contacted for follow-up. The patient reported that she had no limitations accomplishing her activities of daily living or occupational duties. She had become a mother after an uneventful pregnancy in November 2019. She had residual pain in the right sacrum that was worse only after strenuous activity. She reported no physical limitations caring for her toddler and was able to lift, run, and walk with minimal discomfort. She took over-the-counter medication only after strenuous activities or exercise. From a quality of life perspective, she reports that she would never have attempted a pregnancy due to the pain and the potential risks associated with her pain medication regimen.

## Discussion

This case highlights the use of dextrose prolotherapy (DPT) to treat chronic pain and improve activities of daily living as well as the quality of life following the successful treatment of ES of the pelvis. In the 1950s, George S. Hackett M.D. found the technique to be very successful for the treatment of ligament and tendon injuries and named the treatment prolotherapy [9]. Regenerative injection (prolotherapy) is the only known non-operative treatment for the repair of connective tissue/ligament and tendon structural insufficiency following acute or overuse injury, and commonly utilizes dextrose, platelet-rich plasma, or stem cells [10]. Injection of platelet-rich plasma and dextrose are the most accessible and cost-effective forms of regenerative injection.

In this case, the healing potential was likely reduced following chemotherapy and radiation to treat the ES. The open surgical biopsy placed additional scar tissue in the region which may have further compromised the patient’s healing capacity. DPT augmented the tissue healing using dextrose and mechanical disruption from the needle once the patient was in remission. An increase in connective tissue strength and size has been demonstrated in repeated, controlled, and blinded animal studies using hypertonic dextrose injection [11]. DPT also has Level B randomized controlled trial (RCT) evidence across more than 10 chronic musculoskeletal pain indications [12]. In addition to an ameliorative effect on connective tissue insufficiency, dextrose also has increasing evidence of a direct analgesic effect through its downregulation of c-fiber activity [13-14]. DPT is often done with one or more treatment sessions, and resolution of pain occurs once joint stability and normal functional biomechanics are restored [15-17].

In the field of cancer rehabilitation, DPT has been most often used to treat scar-related pain and shoulder/upper body pain in patients in remission from breast cancer. The treatment of patients with cancer after initial chemotherapy or radiation therapy to alleviate pain has been spoken of favorably over the years at annual conferences of the Hackett Hemwall Patterson Foundation, but there has never been a case review according to K.D. Reeves (personal communication, January 1, 2021). In this patient, DPT was used in lieu of more aggressive regenerative treatments to minimize the potential reactivation of the carcinoma. Although all human cells, including tumor cells, utilize glucose for energy, the concentration of glucose injected is expected to drop rapidly due to normal dextrose uptake within local tissues, resulting in minimal regional glucose elevation that may cause tumor growth. Nonetheless, a DPT injection into an untreated or active cancer locus is not recommended. A review of the English language literature found in PubMed did not yield any articles that documented the treatment of cancer-related pain with prolotherapy.

Although many survivors of ES report excellent functional and quality of life outcomes, females and those with pelvic primary tumors are more likely to report significant long-term disability and impairments [5]. Prolotherapy fulfills the do-no-harm principle and gives clinicians a potential tool to impact a patient’s quality of life significantly without any evidence of shortening its length according to K.D. Reeves (personal communication, January 1, 2021). Prolotherapy is a welcome addition to a clinician's toolkit to help improve a patient’s activities of daily living and overall quality of life.

## Conclusions

This is the first known paper to describe the use of dextrose prolotherapy (DPT) to treat sacroiliac region pain following successful treatment of Ewing’s sarcoma. A review of the English language literature found in PubMed did not yield any articles that documented the treatment of cancer-related pain with DPT. Clinicians may consider the use of DPT as a potential treatment option for sacroiliac joint pain and dysfunction following the successful treatment of Ewing's sarcoma of the pelvis to improve a patient's activities of daily living and overall quality of life.
